# High percentages of peripheral blood T-cell activation in childhood Hodgkin's lymphoma are associated with inferior outcome

**DOI:** 10.3389/fmed.2022.955373

**Published:** 2022-08-10

**Authors:** Fengqing Cai, Hui Gao, Zhongsheng Yu, Kun Zhu, Weizhong Gu, Xiaoping Guo, Xiaojun Xu, Hongqiang Shen, Qiang Shu

**Affiliations:** ^1^Department of Clinical Laboratory, The Children's Hospital, National Clinical Research Center for Child Health, Zhejiang University School of Medicine, Hangzhou, China; ^2^Department of Pathology, The Children's Hospital, National Clinical Research Center for Child Health, Zhejiang University School of Medicine, Hangzhou, China; ^3^Department of Hematology-Oncology, The Children's Hospital, National Clinical Research Center for Child Health, Zhejiang University School of Medicine, Hangzhou, China; ^4^National Clinical Research Center for Child Health, The Children's Hospital, Zhejiang University School of Medicine, Hangzhou, China; ^5^Department of Molecular Genetics, University of Toronto, Toronto, ON, Canada

**Keywords:** CD3+CD4+HLA-DR+ T cell, CD3+CD8+HLA-DR+ T cell, risk-stratification, inferior outcome, Hodgkin's lymphoma (HL)

## Abstract

The aims of this study were to investigate the activation of T lymphocytes in peripheral blood from children with Hodgkin's lymphoma (HL) and explore their roles for prognosis in HL. A cohort of 52 newly diagnosed children with HL during the past 10 years was enrolled for analysis in this study. Peripheral blood samples of the patients were acquired before treatment in our hospital, and T-cell subsets were detected by a four-color flow cytometer. CD4+ T cells and CD4+/CD8+ T-cell ratio decreased significantly in patients with HL vs. healthy controls. CD8+ T cells, CD3+CD4+HLA-DR+ T cells, and CD3+CD8+HLA-DR+ T cells increased markedly in patients with HL vs. healthy controls. Receiver-operating characteristic (ROC) curve analysis showed that CD3+CD4+HLA-DR+ T cells and CD3+CD8+HLA-DR+ T cells each distinguished the high-risk group from the low- and intermediate-risk group. The area under the ROC curve for predicting high-risk patients was 0.795 for CD3+CD4+HLA-DR+ T cell and 0.784 for CD3+CD8+HLA-DR+ T cell. A comparison of peripheral blood T-cell subsets that responded differently to therapy showed significantly higher percentages of CD3+CD4+HLA-DR+ T cells and CD3+CD8+HLA-DR+ T cells in patients who achieved complete remission compared to those who did not achieve complete remission. In addition, high percentages of both CD3+CD4+HLA-DR+ T cells and CD3+CD8+HLA-DR+ T cells were associated with inferior event-free survival. Peripheral immune status may be related to disease severity in HL. CD3+CD4+HLA-DR+ T cells and CD3+CD8+HLA-DR+ T cells may be a novel indicator for risk stratification of HL and may be an independent risk factor for inferior outcome in childhood HL.

## Introduction

Hodgkin's lymphoma (HL) is one of the common cancers worldwide, affecting approximately 6,900 new cases every year in China (1, 2). The survival rate of children with HL has been rising gradually during the past three decades and currently exceeds 90% after standard-of-care therapy (2–4). Therefore, HL management has been focused on maintaining cure rate and minimizing late effects according to risk stratifications (5, 6). The Ann Arbor classification of HL was the standard for staging; however, this classification was insufficient for risk stratification before treatment owing to the presence of B symptoms, involved lymph nodes, and extranodal disease. It was important to perform risk-adapted interventions based on a gradual management regime (4, 7, 8). A risk-adapted response-based model for imaging surveillance was developed on the grounds of pretreatment risk stratification and the initial response to therapy (9).

The etiology of HL is unknown, but risk factors that have been identified include previous infection with Epstein–Barr virus (EBV), immune system disorders, and exposure to air pollution (10–14). Rare malignant Hodgkin and Reed–Sternberg (HRS) cells in pathological tissue of HL are encircled by an abundant but ineffective inflammatory cell infiltrate. This unusual feature suggests malignant HRS cells can escape immunosurveillance and be related to B7-1 (CD80), B7-2 (CD86), and PD-1 ligands (15–17). Recent studies of the tumor microenvironment in HL may play an important role and HLA-DR+/CD38 T cells may be related to relapse and refractoriness in pediatric HL (18). Similarly, CD25, the interleukin-2 receptor α-subunit, was expressed on Reed–Stemberg cells and lymphocytes in the tumor environment in HL (19). One study showed that CD69+ cells increased in the tumor cell area in CD4+ or CD8+ T cells (20). However, whether the function of the immune microenvironment in peripheral blood, especially T cells, promoted or restrained HL growth remains to be determined.

This study aims to investigate T-lymphocyte subsets (CD4+, CD8+) and T-lymphocyte activation (CD69+, CD25+, and HLA-DR+) in peripheral blood from children with HL to identify the potential prognostic factors for event-free survival (EFS). We also conducted a pilot investigation about their roles in risk stratification of HL.

## Methods

### Participants

Newly diagnosed children with HL from May 2009 to May 2019 at the Children's Hospital of Zhejiang University School of Medicine were included in this retrospective analysis. The study was approved by the ethics committee of the Children's Hospital of Zhejiang University School of Medicine, and the guardians of all children signed informed consent.

The diagnosis of HL in children was reviewed and confirmed by morphological features and immunohistochemical stains of biopsy samples according to the WHO Classification (21). According to Cotswolds staging classification and risk factors before treatment (22, 23), these patients were divided into two groups, namely, (1) low- and intermediate-risk group (L-I-risk group) (early-stage favorable or unfavorable): patients in stagesIand IIA without or with ≥1 adverse risk factors and stage III without systemic B symptoms or unfavorable features; (2) high-risk group (H-risk group) (advanced stage): patients in stages IIIB and IV or with systemic B symptoms or unfavorable features, including bulky mediastinal masses, extranodal disease, large nodal mass, or more than three nodal sites. Two experienced pathologists and physicians doubly confirmed the diagnosis and grouping. Clinical data of all patients with HL were obtained from our hospital's electronic medical records. Chemotherapy was administered according to the guidelines for treatment of childhood HL (24, 25). Evaluation after chemotherapy was based on the following Lugano-2014 evaluation standard (26): (1) complete remission (CR) was defined as that target lymph node masses must regress < 1.5 cm, no extralymphatic sites of disease, and no new lesions and (2) non-complete remission (NCR), including partial remission, disease stable, and disease progression.

We screened a total of 65 newly diagnosed children with HL during 10 years at the Children's Hospital of Zhejiang University School of Medicine, of which 13 cases were excluded owing to deficiency of medical records (four cases) or no T-cell subset/activation analysis (nine cases). A total of 52 children with HL were finally included in the study for risk assessment. Patients consisted of 44 boys and 8 girls, ranging in age from 2.58 to 15.92 years (median, 8.54 years). Healthy children from our medical center for routine physical examination were included as normal controls. The median age of 30 healthy controls was 9.12 years, ranging from 3.42 to 14.75 years, and 27 cases were boys. There were no significant differences in age and gender between patients with HL and healthy controls.

**Figure 1:**
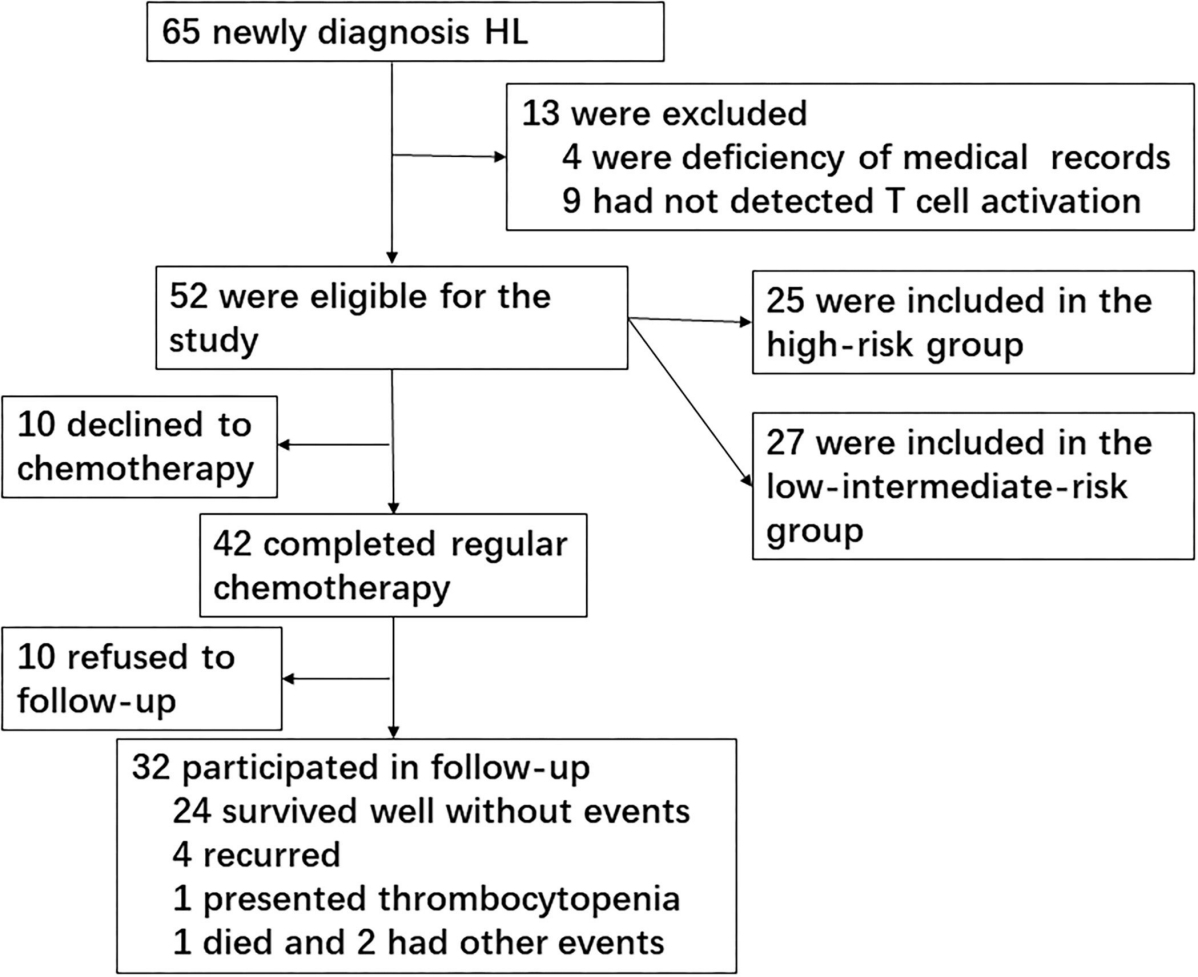
Flowchart screening cases.

Because this was a retrospective study, 10 cases declined the prescription of chemotherapy and went to other hospital seeking treatment; thus, 42 patients completed the chemotherapy course. Of these patients, 29 cases reached CR and 13 cases did not achieve CR (11 cases reached partial remission and two cases presented progressive disease). Finally, 32 patients participated in the follow-up after chemotherapy since 10 patients refused to follow-up. The primary end point was EFS, which was defined as relapse, second cancer, progressive disease, or death from any cause, and the date of last follow-up for children who did not undergo any event. The median follow-up period was 36 months (Figure 1).

### Measurement of T-cell subsets and cytokines

Peripheral blood samples of patients were acquired before treatment in our hospital. A tube of whole blood, which was collected into the EDTA anticoagulation tube, was sent to the laboratory for testing T-cell subsets immediately or within 12 h (the sample was stored at room temperature). Another tube of blood sample was collected into a serum separating tube and centrifuged at 1,000g for 20 min at room temperature after clotting. The serum was harvested carefully for testing cytokines immediately or the test was performed within 12 h if the situation was not so urgent.

The T-cell subsets were detected with a four-color flow cytometer (FACSCalibur™; Becton Dickinson, San Jose, CA). The main reagents used in the study were from BD Biosciences, including CD3-FITC, CD4-APC, CD8-PerCP, CD25-PE, CD69-PE, HLA-DR-PE, PE-Mouse IgG1 Isotype Control, and Lysing Buffer Solution. Briefly, 100 μl peripheral blood was mixed with antibodies (20 μl per antibody) and incubated at 4°C for 30 min in dark, and isotype control was performed at the same time. After incubation, each sample was dissolved with lysing solution and then washed twice with phosphate-buffered saline. Then, each sample was fixed with 1% paraformaldehyde and detected by flow cytometry. 10,000 events were acquired using the CellQuest (version 3.2) software on a FACSCalibur flow cytometer. Gating strategies for the identification of T-cell activation markers were as follows: CD3-positive cells were first gated in the cytogram (R1 gate), then CD4- or CD8-positive cells were gated (R2 gate, refers to R1). Later, CD25-, CD69-, and HLA-DR-positive populations in CD4 or CD8 cells were calculated, respectively (Supplementary Figures S1, S2).

The concentrations of interleukin (IL)-6, IL-10, IL-2, IL-4, tumor necrosis factor alpha (TNF-α), and interferon gamma (IFN-γ) were detected using a CBA kit (BD CBA Human 1/2 Cytokine Kit II; BD Biosciences, San Jose, CA, USA). In short, six bead populations with distinct fluorescence intensities based on CBA technology had been coated with capture antibodies against IL-6, IL-10, IL-2, IL-4, TNF-α, and IFN-γ proteins. Cytokine capture beads were mixed with phycoerythrin-binding detection antibodies, and then incubated with recombinant standards or test samples to form sandwich complexes, which were detected with a flow cytometer (FACSCalibur™; Becton Dickinson, San Jose, CA).

### Statistical analysis

The descriptive data are represented by median and range for continuous variables and percentages for categorical variables. The positive rate is expressed as a percentage (positive cases/total cases tested). The comparison of variables for three groups was performed by Kruskal–Wallis tests, and comparisons between two groups were performed by Mann–Whitney *U-*tests. Comparisons of categorical variables between the two groups were performed by chi-square test. A receiver-operating characteristic (ROC) curve was used to assess whether activated T cells discriminated the H-risk group from the L-I-risk group in HL. Univariate logistic regression analysis was used to estimate odds ratios and 95% confidence interval (CI) for identifying the indicators associated with CR. EFS was estimated by means of the Kaplan–Meier method, and differences in EFS were tested using the log-rank method. A *p* < 0.05 was considered statistically significant. All statistical analyses were performed using IBM SPSS Statistics for Windows, version 26.0 (IBM corp., Armonk, NY, USA). The figures were generated using GraphPad Prism (version 9.0, La Jolla, CA, USA).

## Results

### Basic characteristics of children with HL

Basic characteristics of patients with HL, including initial symptoms, stage, organ involvement, and pathological type, are shown in Table 1. According to Cotswolds staging classification and risk stratification criterion, patients were divided into two groups, namely, L-I-risk group (27 cases, 51.9%) and H-risk group (25 cases, 48.1%), respectively. A total of 52 cases met classic HL type based on morphology and immunophenotype of the neoplastic cells and on the background cellular infiltrate, consisting of 18 cases of NS subtype (34.6%), 25 cases of MC subtype (40.1%), and nine cases of LRC subtype (17.3%). In the H-risk group, predominant patients presented NS subtype (64.0%, 16/25). However, MC subtype prevailed in the L-I-risk group (70.4%, 19/27).

**Table 1:** Clinical characteristics of children with HL.

**Characteristic**	**Whole group** **(*n* = 52)**	**H-risk group** **(*n* = 25)**	**L-I-risk group** **(*n* = 27)**	***P***
**Ages (years)**				NS
Median	8.54	8.33	8.95	
Range	2.58–15.92	2.58–15.92	4.67–14.50	
**Gender**				NS
Boys	44	20	24	
Girls	8	5	3	
**Initial symptoms**				0.013
Cervical lymphadenopathy	42 (80.7%)	17 (32.7%)	25 (40.1%)	
Mediastinal lymph node enlargement	2 (3.8%)	2 (3.8%)	0 (0.0%)	
Both cervical lymphadenopathy and mediastinal lymph node enlargement	4 (7.7%)	4 (7.7%)	0 (0.0%)	
Other lymphadenopathies	2 (3.8%)	0 (0.0 %)	2 (3.8%)	
Abnormal gait with right lower limb	1 (1.9%)	1 (1.9%)	0 (0.0%)	
Anemia with fever	1 (1.9%)	1 (1.9%)	0 (0.0%)	
**Ann Arbor stage**				<0.001
Stage I	3 (5.8%)	0 (0.0 %)	3 (5.8%)	
Stage II	14 (26.9%)	0 (0.0 %)	14 (26.9%)	
Stage III	21 (40.4%)	11 (21.2 %)	10 (19.2%)	
Stage IV	14 (26.9 %)	14 (26.9 %)	0 (0.0 %)	
B Symptoms	19 (36.5 %)	19 (36.5%)	0 (0.0 %)	
**Organ involvement**				<0.001
Liver	17 (32.7%)	14 (26.9%)	3 (5.7%)	
Spleen	19 (36.5%)	16 (30.8%)	2 (3.5%)	
**Pathological type**				<0.001
NS	18 (34.6%)	16 (30.8%)	2 (3.8%)	
MC	25 (40.1%)	6 (11.5%)	19 (36.5%)	
RC	9 (17.3%)	3 (5.8%)	6 (11.5%)	
**EBV infection**				
EBV-DNA (+) ^a^ (n = 27)	16 (59.3 %)	12 (44.4 %)	4 (14.8 %)	0.014
EBER (+) /LMP (+) ^b^ (n = 25)	12 (48.0 %)	3 (12.0 %)	9 (36.0 %)	0.271

*NS, nodular sclerosis; MC, mixed cellularity; LRC, lymphocyte-rich classic; a: variable in blood or bone marrow; b: variable in biopsy. Data was expressed as the numbers (%)*.

A study of EBV infection in patients showed that the 25 cases among 52 patients who received immunohistochemical staining for Epstein–Barr encoded RNA (EBER) or EBV-latent membrane protein (LMP) in biopsy specimens included 12 positive cases (48.0%) and 13 negative cases (52.0%). A total of 27 patients had EBV-DNA examined in blood or bone marrow, and included 16 cases (59.3%) with high EBV viral load. Statistical analysis showed significantly higher EBV-DNA-positive rate in the H-risk group than in the L-I-risk group (Table 1).

### Peripheral blood T-cell subsets and serum cytokines in patients with HL

**Figure 2:**
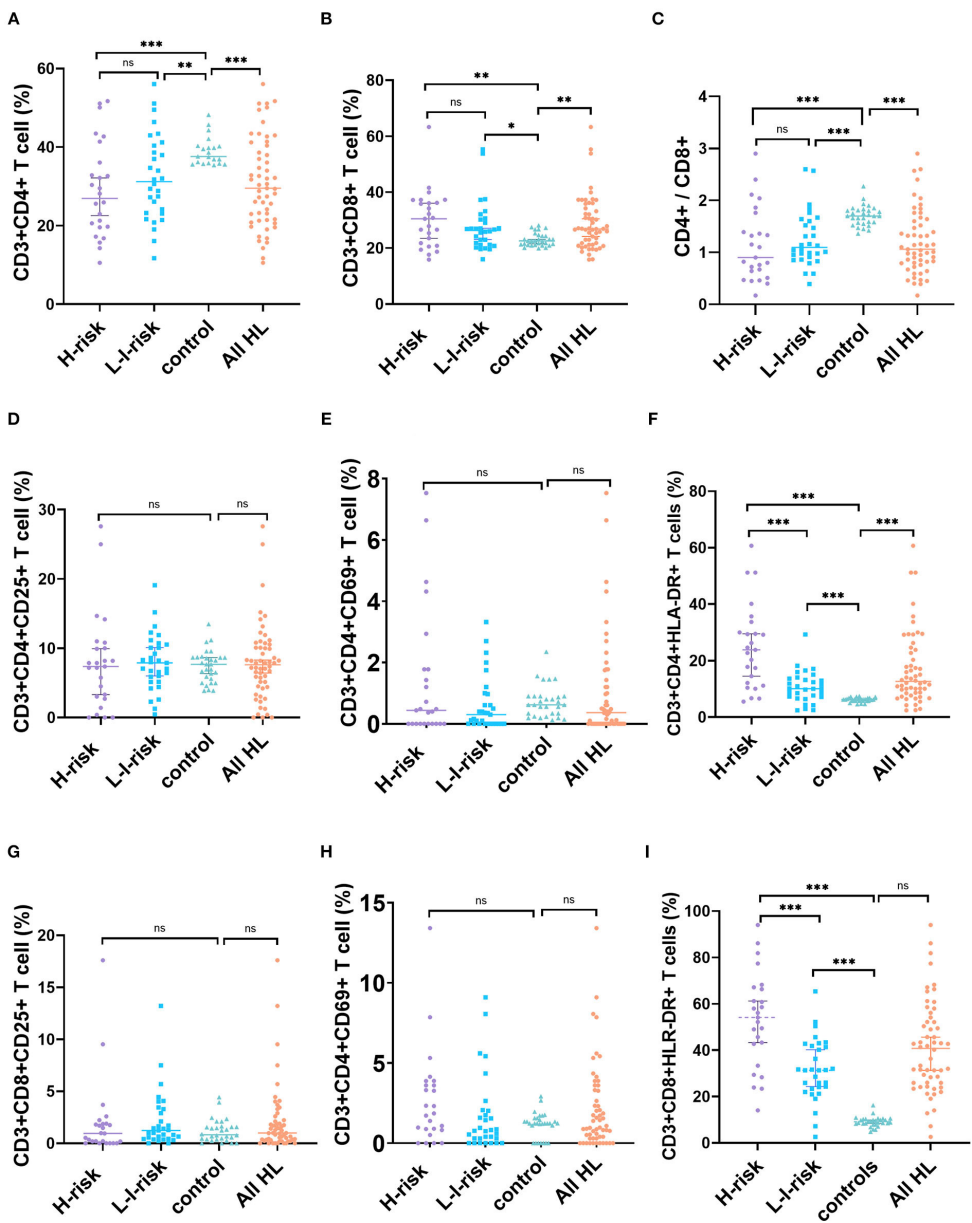
Changes in percentages of T-cell subset markers in patients with HL and health controls. **(A)** CD4+ T cells, **(B)** CD8+ T cells, **(C)** CD4+/CD8+ ratio, **(D)** CD3+CD4+CD25+ T cells, **(E)** CD3+CD4+CD69+ T cells, **(F)** CD3+CD4+HLA-DR+ T cells, **(G)** CD3+CD8+CD25+ T cells, **(H)** CD3+CD8+CD69+ T cells, and **(I)** CD3+CD8+HLA-DR+ T cells. ns, not significant, *p* > 0.05; *p < 0.05, **p < 0.01; ***p < 0.001.

The percentage of CD4+ T cells (median, 28.82%, range, 10.55–56.07%, Z = −3.912, *p* = 0.001) and CD4+/CD8+ ratio (median, 1.01, range, 0.17–2.90, Z = −4.846, *p* < 0.001) decreased significantly in patients with HL compared to healthy controls. The percentage of CD8+T cells (median, 26.90%, range, 15.89–63.31%, Z = 2.983, *p* = 0.003) increased markedly in patients with vs. healthy controls. However, there was no statistically significant difference in the percentage of T cell subsets, including CD3+CD4+ T cell, CD3+CD8+ T cell, and CD3+CD4+/CD3+CD8+ T cell ratio in two risk classification groups (Figure 2; Supplementary Table 1).

We further identified T-cell activation as a critical part of pathogenesis of patients with HL. In peripheral blood, the percentage of CD3+CD4+HLA-DR+ T cells increased significantly in patients with HL (median, 13.40%, range, 2.90–60.71%) vs. healthy controls (median, 6.25%, range, 4.56–7.45%, Z = 6.065, *p* < 0.001), and the percentage of CD3+CD8+HLA-DR+ T cells elevated markedly in patients with HL (median, 42.26%, range, 2.60–93.98%) vs. healthy controls (median, 9.08%, range, 4.76–16.20%, Z = 7.211, *p* < 0.001). In contrast, no statistically significant difference was found in the percentage of CD3+CD4+CD25+ T cells, CD3+CD4+CD69+ T cells, CD3+CD8+CD25+ T cells, or CD3+CD8+CD69+ T cells between HL and healthy controls. This result indicated that children with HL had higher expressions of CD3+CD4+HLA-DR+ T cells and CD3+CD8+HLA-DR+ T cells than those in healthy controls. Interestingly, a comparison of the percentage of HLA-DR+ T cells in the peripheral blood of different risk patients showed a significantly higher percentage of CD3+CD4+HLA-DR+ T cells (Z = −3.645, *p* < 0.001) and CD3+CD8+HLA-DR+ T cells (Z = −3.507, *p* < 0.001) in the H-risk group compared to those in the L-I-risk group.

Because activated T cells can secrete a mass of cytokines that play a crucial part in antitumor immune response, we analyzed cytokine levels (IL-2, IL-4, IL-6, IL-10, TNF-α, and IFN-γ in children with HL). The levels of IL-6 (Z = 3.900, *p* < 0.001) and IL-10 (Z = 5.661, *p* < 0.001) in patients with HL were significantly increased compared to the control group. There were no significant differences in the levels of IL-2, IL-4, TNF-α, or IFN-γ between patients with HL and normal controls. Additionally, the levels of IL-2 (Z = 2.081, *p* = 0.037), IL-6 (Z = 3.719, *p* < 0.001), IL-10 (Z = 3.132, *p* = 0.002), and IFN-γ (Z = 2.134, *p* = 0.033) of patients in the H-risk group were significantly higher than those in the L-I-risk group (Figure 2; Supplementary Table 1).

### The value of activated T cells and serum cytokines in risk classification of HL

**Figure 3:**
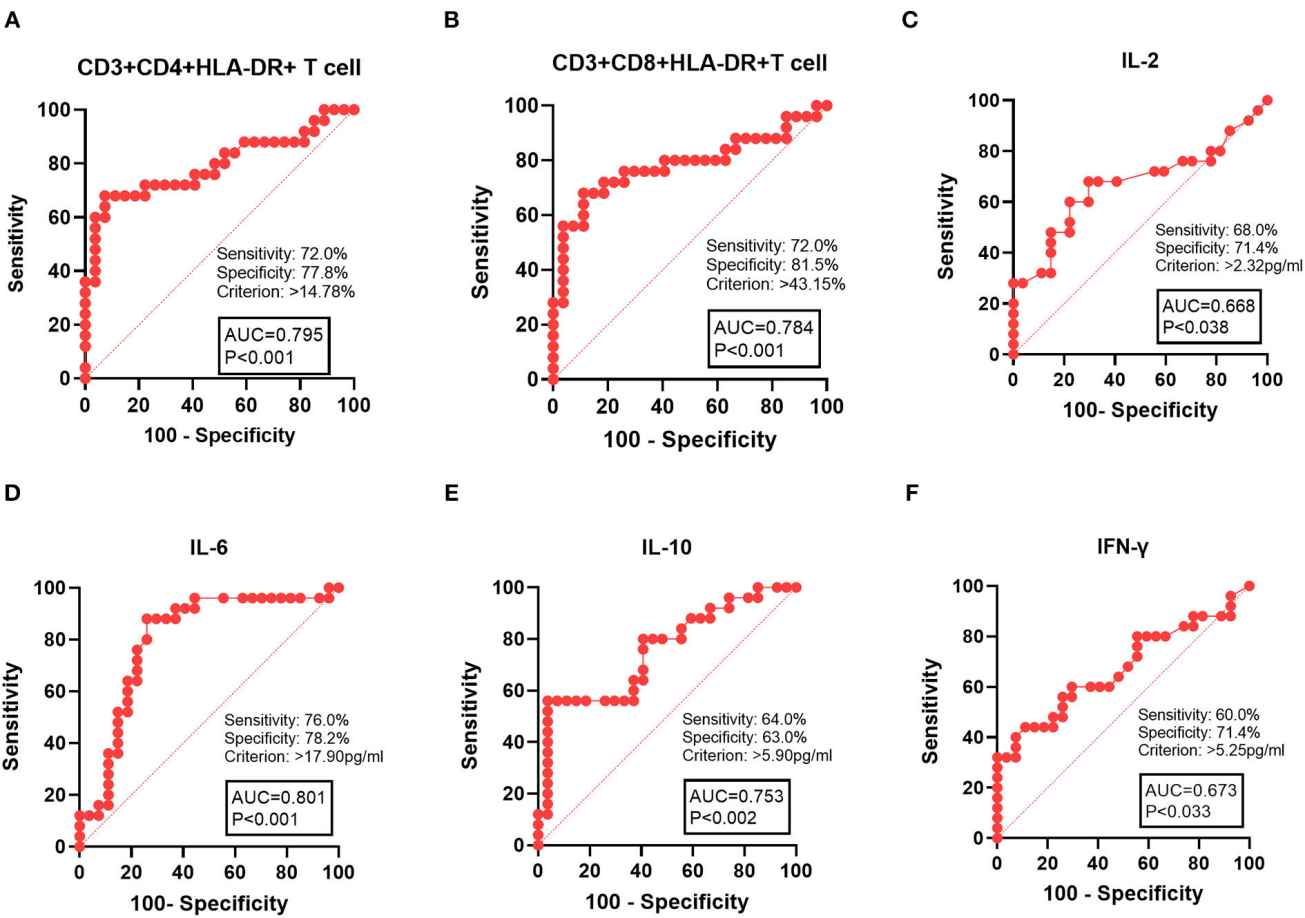
ROC curve analysis of laboratory indices with patients with HL in the H-risk group. **(A)** CD3+CD4+HLA-DR+ T cells, **(B)** CD3+CD4+HLA-DR+ T cells, **(C)** IL-10, **(D)** IL-6, **(E)**, IL-10, and **(F)** IFN-γ. AUC, area under the ROC curve.

To further assess the role of T-cell activation in disease severity of HL, we analyzed CD3+CD4+HLA-DR+ and CD3+CD8+HLA-DR+ T cells in two risk classification groups. Compared to the L-I-risk group, laboratory indices were found to differ significantly in the H-risk group, including CD3+CD4+HLA-DR+ T cells, CD3+CD8+HLA-DR+ T cells, IL-2, IL-6, IL-10, and IFN-γ. Therefore, these laboratory indices could be used for risk classification in HL. We analyzed ROC for CD3+CD4+HLA-DR+ T cells, CD3+CD8+HLA-DR+ T cells, HB, IL-2, IL-6, IL-10, and IFN-γ, which were used to distinguish the H-risk group from the L-I-risk group. The area under the ROC curve (AUC) for predicting H-risk patients was 0.795 for CD3+CD4+HLA-DR+ T cell, 0.784 for CD3+CD8+HLA-DR+ T cell, 0.801 for IL-6, 0.753 for IL-10, 0.673 for IFN-γ, and 0.668 for IL-2. The maximum efficiency was determined with a cutoff value of 14.78% based on CD3+CD4+HLA-DR+ T-cell percentage, offering a sensitivity of 72.0% and specificity of 77.8%. The maximum efficiency was determined with a cutoff value of 43.15% based on CD3+CD8+HLA-DR+ T-cell percentage, offering a sensitivity of 72.0% and specificity of 81.5% (Figure 3).

### Comparisons of laboratory indices between CR and NCR after chemotherapy

In 42 patients who completed chemotherapy course, 29 reached CR and 13 did not achieve CR (11 cases reached partial remission and two cases presented with progressive disease). A comparison of peripheral blood T-cell subsets and serum cytokines in patients who responded differently to therapy showed significantly higher values of CD3+CD4+HLA-DR+ T cells (Z = 4.149, *p* < 0.001), CD3+CD8+HLA-DR+ T cells (Z = 4.666, *p* < 0.001) and IL-6 (Z = 2.354, *p* = 0.019) in patients who achieved CR compared to those who did not achieve CR. To further investigate the significance of HLA-DR+ T cells and IL-6 in patients who achieved CR, univariate analyses were performed to identify prognostic factors. These parameters, including CD3+CD4+HLA-DR+ T cells (odds ratio [OR] =1.190, 95% CI 1.067–1.328, *p* = 0.002) and CD3+CD8+HLA-DR+ T cells (OR = 1.235, 95% CI 1.071–1.426, *p* = 0.004), were associated with NCR in HL (Tables 2, 3).

**Table 2:** Comparisons of indicators between CR and non-CR group.

**Parameters**	**CR (*N* = 29)**	**NCR (*N* = 13)**	**Z**	***P***
CD3+CD4+ T cell (%)	29.53 (19.05–49.50)	41.4 (14.29–56.07)	1.129	0.268
CD3+CD8+ T cell (%)	26.94 (15.89–55.29)	25.36 (15.98–41.57)	−−0.775	0.438
CD4+ T cell/CD8+ T cell	0.98 (0.39–1.92)	1.33 (0.40–2.90)	1.114	0.265
CD3+CD4+CD25+ T cell (%)	7.05 (0.46–25.00)	7.36 (0.00–27.59)	−−0.312	0.755
CD3+CD4+HLA–DR+ T cell (%)	10.20 (2.40–35.71)	26.26 (10.40–60.41)	4.149	<0.001
CD3+CD4+CD69+ T cell (%)	0.36 (0.00–2.94)	0.48 (0.00–7.53)	0.305	0.760
CD3+CD8+CD25+ T cell (%)	0.98 (0.00–13.21)	1.70 (0.00–17.60)	0.908	0.364
CD3+CD8+HLA–DR+ T cell (%)	31.58 (2.60–57.94)	60.80 (45.60–86.12)	4.666	<0.001
CD3+CD8+CD69+ T cell (%)	0.82 (0.00–9.10)	1.86 (0.00–7.86)	1.113	0.266
IL–2 (pg/ml)	2.20 (1.00–4.70)	2.40(1.10–6.80)	−−0.354	0.723
IL–4 (pg/ml)	2.50 (0.80–12.30)	2.50 (0.80–7.10)	−−0.286	0.775
IL–6 (pg/ml)	7.70 (1.70–189.00)	60.30 (1.40–2068.30)	2.354	0.019
IL–10 (pg/ml)	5.30 (1.80–49.80)	13.60 (2.00–163.00)	1.837	0.068
TNF–α (pg/ml)	2.20 (1.00–14.20)	2.20 (1.00–18.30)	−−0.068	0.946
IFN–γ (pg/ml)	4.20 (1.00–24.00)	6.80 (1.30–53.40)	1.306	0.191

*WBC, white blood cell count; HB, hemoglobin; PLT, platelet count; hs-CRP, hypersensitive C-reactive protein; IL, interleukin; TNF-α, tumor necrosis factor-α; IFN-γ, interferon-γ. Values were the median (range)*.

**Table 3:** Logistic regression analysis for identifying independent indicator associated with complete remission in HL.

**Parameters**	**B**	**S.E**.	**Wals**	***P* value**	**Odds ratio**	**95%CI**	
CD3+CD4+HLA-DR+ T cell (%)	0.174	0.056	9.803	0.002	1.190	1.067	1.328
CD3+CD8+HLA-DR+ T cell (%)	0.211	0.073	8.314	0.004	1.235	1.071	1.426
IL-6 (pg/ml)	0.014	0.007	3.488	0.064	1.014	0.999	1.029

*HB, hemoglobin; hs-CRP, hypersensitive C-reactive protein; IL, interleukin*.

### Relationship between T-cell activation and event-free survival

**Figure 4:**
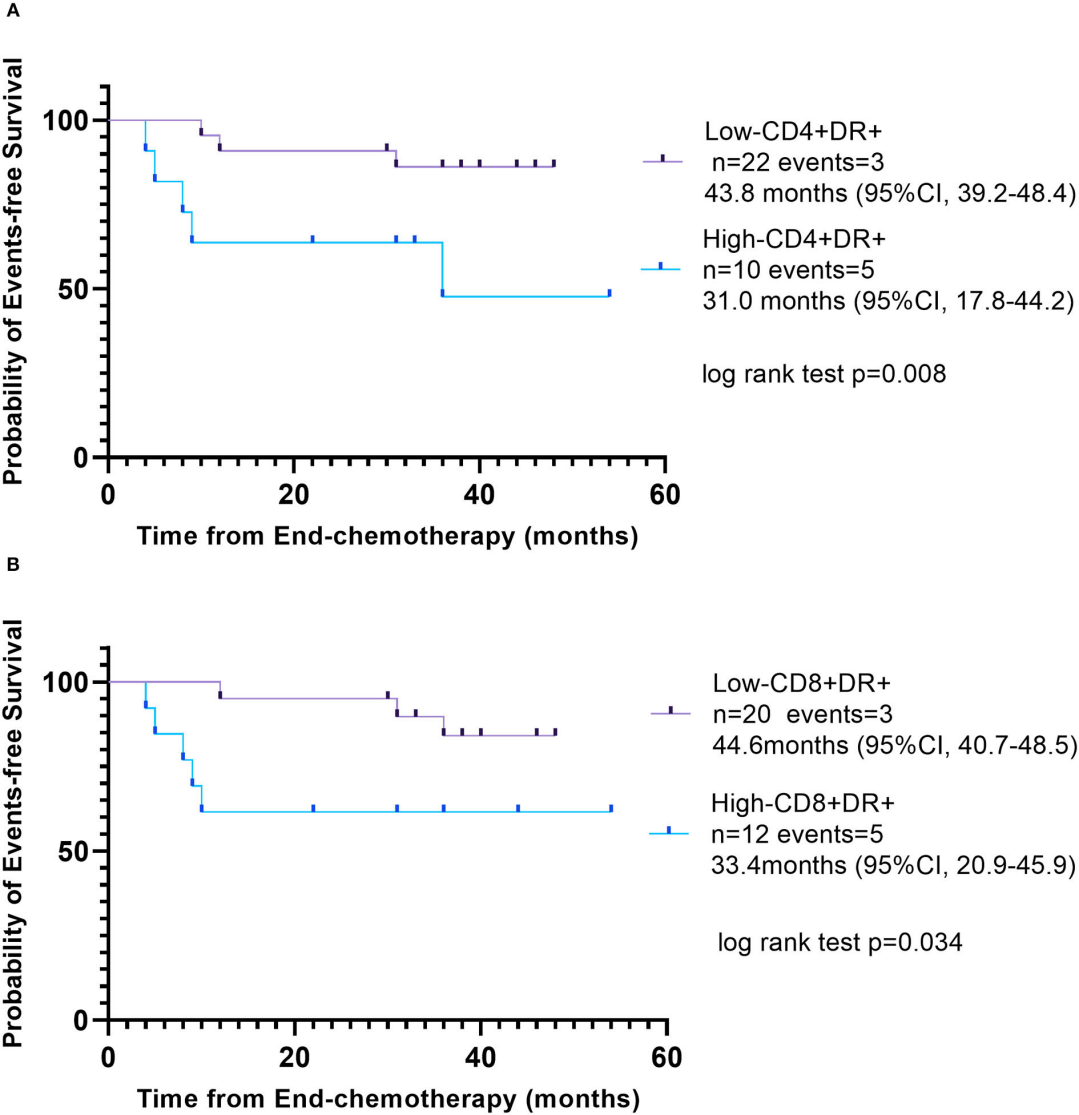
Event-free survival according to different percentage of activated T cells. **(A)** Low CD3+CD4+HLA-DR+ T cell (percentage ≤ 14.78%), purple line and high CD3+CD4+HLA-DR+ T cell (percentage >14.78%), blue line. **(B)** Low CD3+CD8+HLA-DR+ T cell (percentage ≤ 43.15%), purple line; high CD3+CD8+HLA-DR+ T cell (percentage >43.15%), blue line.

In the follow-up, 32 patients finally participated in this study after chemotherapy (owing to 10 patients refusing to follow-up). The primary end point was EFS, which was defined as relapse, second cancer, progressive disease, or death from any cause, and the date of last follow-up for children who did not undergo any event. The median follow-up period was 36 months. Kaplan–Meier analysis showed a significant difference between patients with low CD3+CD4+HLA-DR+ T cell (percentage ≤ 14.78%) and high CD3+CD4+HLA-DR+ T cell (percentage > 14.78%). Kaplan–Meier analysis also showed a significant difference between patients with low CD3+CD8+HLA-DR+ T cell (percentage ≤ 43.15%) and high CD3+CD8+HLA-DR+ T cell (percentage > 43.15%). High percentages of CD3+CD4+HLA-DR+ T cell (*p* = 0.008, log-rank test) and CD3+CD8+HLA-DR+ T cell (*p* = 0.034, log-rank test) were associated with the inferior EFS (Figure 4).

**Figure 5:**
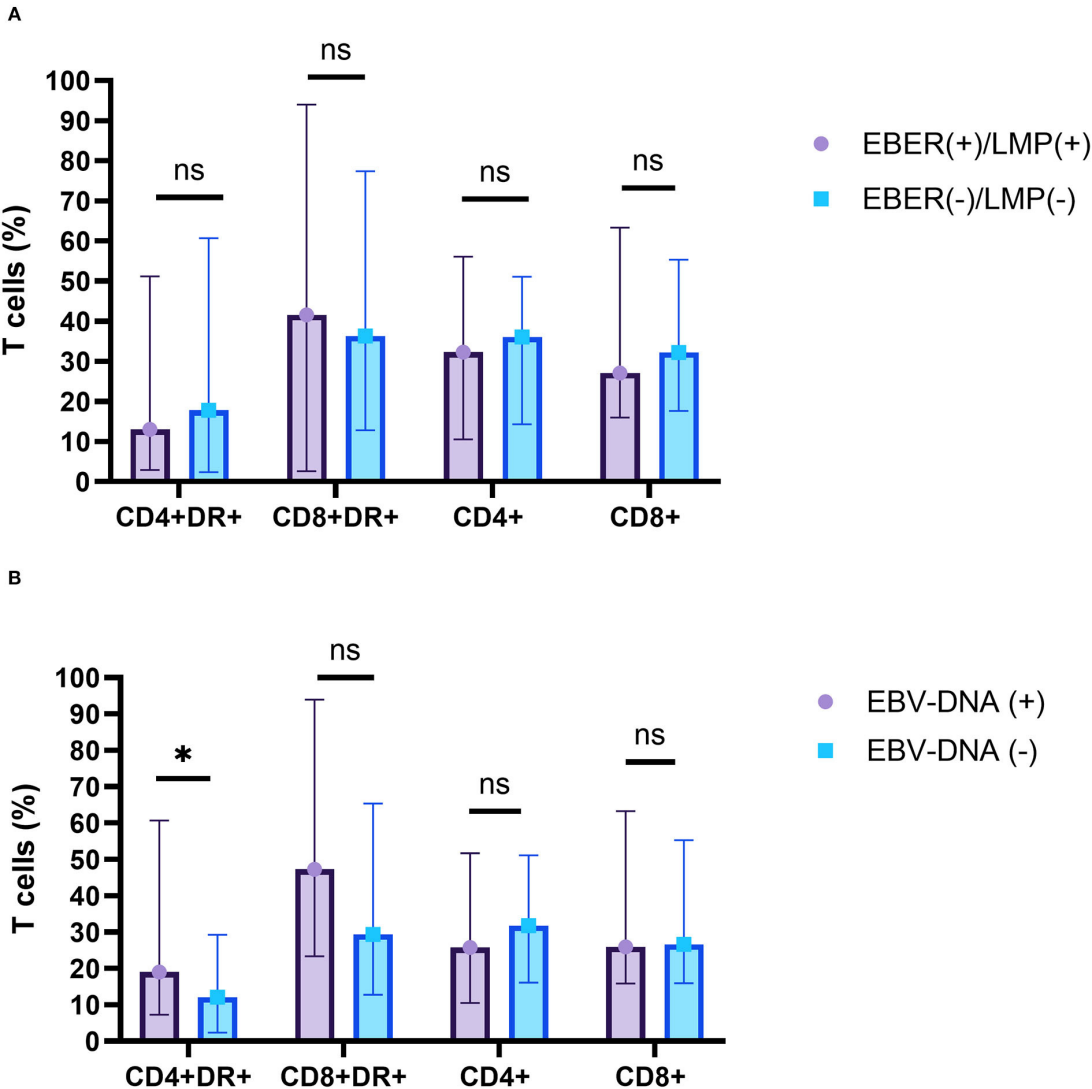
The relationship between the percentage of T-cell subsets and EBV infections. **(A)** EBV-EBER- or LMP-1-positive patients (purple) and EBV-EBER- or LMP-1-negative patients (blue) in biopsy specimens. **(B)** EBV-DNA-positive patients (purple) and EBV-DNA-negative patients (blue) in serum. ns, not significant, *p* > 0.05; *p < 0.05.

Many studies demonstrated that EBV present in HRS cells in 40–50% of HL cases and may be involved in the pathogenic mechanism of tumorigenesis. This study found that EBER or LMP positive in biopsy specimens was detected in 48.0% (12/25) of patients and that EBV-DNA positive in blood or bone marrow was detected in 59.3% (16/27) of patients. However, there was no significant difference in the percentage of CD4+, CD8+, CD3+CD4+HLA-DR+ T cells, or CD3+CD8+HLA-DR+ T cells in peripheral blood between EBER- or LMP-positive patients and EBER- or LMP-negative patients in biopsy specimens. The percentage of CD3+CD4+HLA-DR+ T cell in peripheral blood was significantly higher in EBV-DNA–positive patients compared to EBV-DNA-negative patients (Z = 2.122, *p* = 0.034). There was no significant difference in the percentage of CD4+, CD8+ and CD3+CD8+HLA-DR+ T cells in peripheral blood between EBV-DNA-positive patients and EBV-DNA–negative patients (Figure 5).

**Figure 6:**
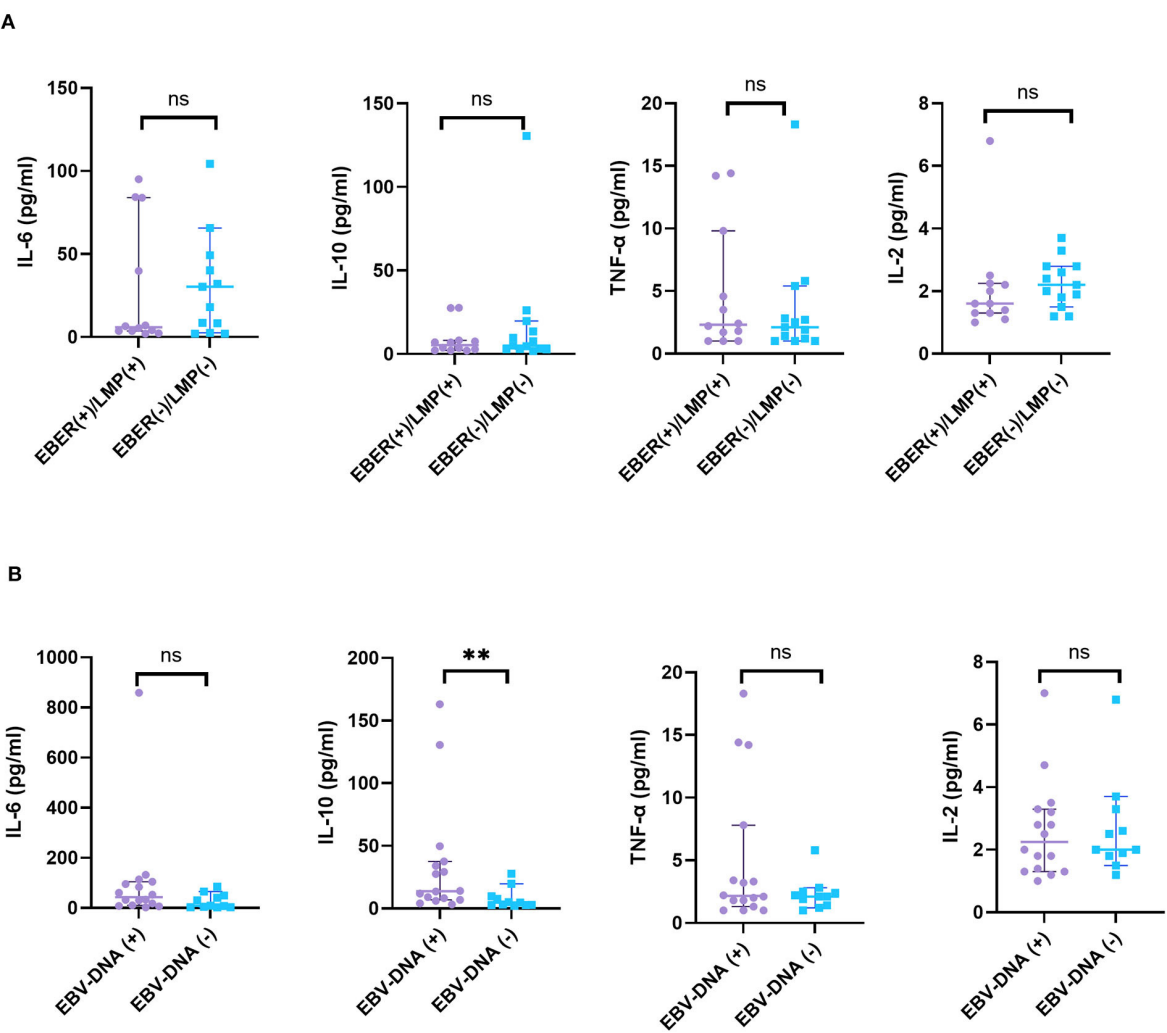
The relationship between cytokines and EBV infections. **(A)** A total of 25 patients with HL were grouped according to the expression of EBER or LMP by HRS cells and **(B)** 27 patients with HL were grouped according to EBV-DNA in blood or bone marrow. ns, not significant, *p* > 0.05; **p < 0.01.

The levels of cytokines were not significantly different in EBER- or LMP–positive and -negative patients with HL. There was also no significant difference in the levels of cytokines, including IL-2, IL-4, IL-6, TNF-α, or IFN-γ between EBV-DNA-positive and EBV-DNA–negative patients. Nevertheless, higher level of IL-10 in peripheral blood was detected in EBV-DNA-positive patients with HL compared to those in EBV-DNA-negative patients with HL (Z = 2.690, *p* = 0.006) (Figure 6).

## Discussion

Diagnosis of HL depends on the discovery of neoplastic Hodgkin's cells and Reed–Sternberg cells in lots of inflammatory background. The neoplastic cells are derived mainly from germinal center B cells that are infected by EBV. Many studies demonstrated that EBV infection was associated with a number of lymphomas. The molecular pathogenesis of HL, which shows a balance of proliferative and apoptotic effects, involves TRAFs, NF-kB, STAT and cytokine pathways, and overexpression of PD-1 with inhibition of caspase activities (27). Expression of EBV markers, including EBV-LMP or EBER, was a valuable finding in classic patients with HL, where EBV expression is characteristically shown in the HRS cells (10, 28). In agreement with those results, our results found that 48.0% cases of HL presented LMP or EBER positivity in biopsy specimens. High EBV-DNA viral load was detected in 44.4% of the H-risk group in our study. In addition, EBV infection may be involved in pathogenic mechanism of tumorigenesis. Lin et al. (10) reported that LMP1 in HL cells can lead a moderate increase in autophagy signals by increasing expression of the autophagy marker LC3, including reduced autophagic stress and alleviated autophagy inhibition-induced cell death. Marshall et al. (29) suggested that EBV-LMP1 epitopes could induce HL-infiltrating lymphocyte Treg cells. However, this study showed that there were no significant differences in the percentage of CD3+CD4+HLA-DR+ T cells, CD3+CD8+HLA-DR+ T cells, CD4+ T cells, and CD8+ T cells between LMP- or EBER-positive patients and LMP- or EBER–negative patients. This result may suggest that T-cell activation in HL was induced by HRS cell, rather than stimulated by EBV infection. The role of activated T cell in the immune microenvironment, whether to promote or to fight against HRS cell growth, remained controversial. HRS cells were embraced by the amount of reactive leucocytes (30). The features suggested that HL tumor cells escaped from immunosurveillance for their survival and growth, including CTLA-4, PD-1, and LAG-3-associated immune evasion (31, 32).

In this study, we hypothesized that CD4+ T cells and CD8+ T cells were involved in the progression of HL. In a previous study, MHC class II on HRS cells was a predictive marker for CR and prolonged progression-free survival in patients with HL after PD-1 blockade, suggesting the importance of CD4+ T cells in the tumor environment of CHL (33). They also found that LAG-3 was highly expressed *via* tumor-infiltrating CD4+ lymphocytes in MHC-II-expressing tumors. In lymph node biopsied of HL, a mass cytometry discovered a CD4+ regulatory T-cell rich and exhausted T effector tumor microenvironment (34). Similarly, Sara et al. demonstrated high CD4+ and low CD8+ T cells in diagnostic biopsies by flow cytometry associated with poor prognosis in patients with HL (35). However, our finding showed lower CD4+ T cells and CD4+/CD8+ ratio, but higher percentage of CD3+CD4+HLA-DR+ T cells and CD3+CD8+HLA-DR+ T cells in peripheral blood of patients with HL vs. healthy controls. Thus, the pathogenesis of HL was related to the immune environment, especially the activated T lymphocytes.

Furthermore, we also studied the percentage of activated T cells in risk classification of HL. Pathological staging had little value in the choice of treatment scheme, and risk grading before treatment was more important. Clinical risk stage remained the most important prognostic factor in HL (28). An *in vitro* study about immune mechanism of CD8+HLA-DR+ T cells showed that a higher expression of PD-1 and TIGIT ligand CD155 was evaluated on day 4 after CD8+HLA-DR+ T-cell activation than those in their CD8+HLA-DR- counterpart (36). They found further that PD-1/PD-L1 pathway suppressed activity on CD4+ T cells or CD8+ T cells, but PD-1 neutralization eliminates the suppression ability on CD8+ cells, with little action on CD4+ T cells. However, CTLA-4 on CD4+ and CD8+ T cells also may be involved in their suppressor (32, 37). We found that peripheral blood percentages of CD3+CD4+HLA-DR+ and CD3+CD8+HLA-DR+ T cells were higher in patients at H-risk group vs. L-I-risk group, and that CD3+CD4+HLA-DR+ and CD3+CD8+HLA-DR+ T cells could distinguish the H-risk group from the L-I-risk group. Our data also revealed an association of low percentages of CD3+CD4+HLA-DR+ T cells and CD3+CD8+HLA-DR+ T cells with CR in HL, which implied that HLA-DR+ T-cell activation may help tumor cells to escape immune surveillance.

Next, we found that high percentages of CD3+CD4+HLA-DR+ T cell and CD3+CD8+HLA-DR+ T cell were associated with the inferior EFS. Consistent with our finding, a review summarized the immune microenvironment in HL regarding T cells and immune checkpoints explicated activated T cells cannot exert antitumor immunity due to chronic ineffectual antigen stimulation by upregulation of PD-1 and inability of T cells to recognize HRS cells (38). Owing to mutations, for instance, in the β*-2M* and *CIITA* genes (39) and by epigenetic mechanisms, HRS cells lack expression of MHC-I and MHC-II molecule, inducing CD4+ T cells and CD8+ T cells unable to receive antigen recognition (31). A multivariate analysis previously showed high proportions of both PD-1+ and PD-L1+ leukocytes of the tumor microenvironment were associated with poor EFS and poor overall survival in CHL (40). A previous study found the high ratio of CD3+/HLA-DR+ T cells, both on CD4+ helper and CD8+ cytotoxic T cells after chemotherapy, may predict the danger of relapse in patients with non-Hodgkin's lymphoma (41).

Meanwhile, the levels of IL-6, IL-10, and IFN-γ were significantly increased in the H-risk group vs. the L-I-risk group. Activation of T lymphocytes stimulated generation of a mass of cytokine storm that included downstream effector molecules. A previous study suggested that IL-6/STAT3 signaling of human DCs would downregulate the surface expression of HLA class II molecules (42). IL-10, a kind of immunosuppressive cytokines, was produced at high levels in tumor-bearing states to restrain the function of antitumor effector T cells (43). IFN-γ production was important in cell-mediated immune responses through p38 mitogen-activated protein kinase pathway (44), and IFN-γ can be secreted by HRS cells to induce expression of PD-L1 (45). Concurrently, we analyzed the levels of cytokines, such as IL-2, IL-4, IL-6, IL-10, TNF-α, and IFN-γ, and there were no significant differences in EBV-EBER- or LMP-1-positive and -negative patients with HL. Therefore, these cytokine expressions may not be yet associated with EBV-EBER- or LMP-1-positive cells in biopsy specimens. There were also no significant differences in the levels of cytokines, including IL-2, IL-4, IL-6, TNF-α, or IFN-γ between EBV-DNA-positive and EBV-DNA-negative patients in peripheral blood or bone marrow. Nevertheless, higher IL-10 levels in peripheral blood were detected in EBV-DNA-positive patients with HL compared to those in EBV-DNA-negative patients with HL. IL-10 secretion in HL can suppresses Th1 responses that could promote the antitumor response and high IL-10 receptor in tumor cells was seen in EBV lymphomagenesis (46, 47). The changes of cytokines may contribute to antitumor immunity evasion.

Nevertheless, there are a few limitations to this study. First, this was a retrospective analysis, so the samples were not detected by the same batch. We controlled the batch difference through daily indoor quality control. Second, the treatment of patients with CHL was guided by risk category. All the patients were divided into three groups, namely, low-risk HL, intermediate-risk HL, and high-risk HL, according to current standards. However, we found that there was no difference in the percentage of T-cell subsets between low- and intermediate-risk HL through pre-analysis. So, we mainly analyzed the differences of T-cell subsets between the H-risk group and L-I-risk group. Third, this study did not analyze T-cell exhaustion marker (PD-1, TIM-3, etc.) before treatment, so the mechanism between T-cell activation and exhaustion cannot be well elaborated. In the future, we will study changes of T cells before and after chemotherapy and explore the relationship between T cell and immune checkpoints.

In conclusion, peripheral immune status may be related to disease severity in HL. CD3+CD4+HLA-DR+ T cells and CD3+CD8+HLA-DR+ T cells may be a novel indicator for risk stratification of HL and may be an independent risk factor for inferior outcome in childhood HL.

## Data availability statement

The original contributions presented in the study are included in the article/Supplementary material, further inquiries can be directed to the corresponding authors.

## Ethics statement

The studies involving human participants were reviewed and approved by the Medical Ethics Committee of The Children's Hospital of Zhejiang University School of Medicine (Hangzhou, China). Written informed consent to participate in this study was provided by the participants' legal guardian/next of kin.

## Author contributions

HS and QS designed the study and contributed equally to this study. FC collected data from the electronic medical record and performed the statistics and wrote the article. HG and ZY checked all the laboratory data and participated in the discussion section, including the literature review. WG and KZ participated in the verification of pathological sections. XG and XX were responsible for sorting out the medical records. All authors approved the final version of the manuscript and agreed with the order of the authors before submission and publication.

## Conflict of interest

The authors declare that the research was conducted in the absence of any commercial or financial relationships that could be construed as a potential conflict of interest.

## Publisher's note

All claims expressed in this article are solely those of the authors and do not necessarily represent those of their affiliated organizations, or those of the publisher, the editors and the reviewers. Any product that may be evaluated in this article, or claim that may be made by its manufacturer, is not guaranteed or endorsed by the publisher.

## References

[1] Liu W Liu J Song Y Zeng X Wang X Mi L. Burden of lymphoma in china, 2006-2016: an analysis of the global burden of disease study 2016. J Hematol Oncol. (2019) 12:115. 10.1186/s13045-019-0785-7 31744509 PMC6862726

[2] Kelly KM Hodgson D Appel B Chen L Cole PD Horton T. Children's oncology group's 2013 blueprint for research: hodgkin lymphoma. Pediatr Blood Cancer. (2013). 60:972–8. 10.1002/pbc.24423 23255501

[3] Hochberg J Cairo MS. Lymphoma in adolescents and young adults: current perspectives. Cancer J. (2018) 24:285–300. 10.1097/PPO.0000000000000345 30480573

[4] Giulino-Roth L Keller FG Hodgson DC Kelly KM. Current approaches in the management of low risk Hodgkin lymphoma in children and adolescents. Br J Haematol. (2015) 169:647–60. 10.1111/bjh.13372 25824371

[5] Lo AC Dieckmann K Pelz T Gallop-Evans E Engenhart-Cabillic R Vordermark D. Pediatric classical Hodgkin lymphoma. Pediatr Blood Cancer. (2021) 68:e28562. 10.1002/pbc.28562 33818890

[6] Bartlett N.L. Protect our children: Hodgkin lymphoma survivors. Blood. (2021) 137:1433–4. 10.1182/blood.2020010320 33734336

[7] Goldman S Smith L Anderson JR Perkins S Harrison L Geyer MB. Rituximab and FAB/LMB 96 chemotherapy in children with Stage III/IV B-cell non-Hodgkin lymphoma: a Children's Oncology Group report. Leukemia. (2013) 27:1174–7. 10.1038/leu.2012.255 22940833 PMC4539148

[8] Gómez-Almaguer D González-Llano O Jiménez-Antolinez V Gómez-De León A. Treatment of classical Hodgkin's lymphoma in children and adolescents. Expert Opin Pharmacother. (2019) 20:1227–34. 10.1080/14656566.2019.1606212 31021660

[9] Voss SD Cairo MS. Surveillance imaging in pediatric lymphoma. Pediat Radiol. (2019) 49:1565–73. 10.1007/s00247-019-04511-4 31620855

[10] Lin HC Chang Y Chen RY Hung LY Chen PC Chen YP. Epstein-Barr virus latent membrane protein-1 upregulates autophagy and promotes viability in Hodgkin lymphoma: Implications for targeted therapy. Cancer Sci. (2021) 112:1589–602. 10.1111/cas.14833 33525055 PMC8019199

[11] Cader FZ Hu XH Goh WL Wienand K Ouyang J Mandato E. A peripheral immune signature of responsiveness to PD-1 blockade in patients with classical Hodgkin lymphoma. Nat Med. (2020) 26:1468–79. 10.1038/s41591-020-1006-1 32778827 PMC9673009

[12] Taj T Poulsen AH Ketzel M Geels C Brandt J Christensen JH. Long-term residential exposure to air pollution and Hodgkin lymphoma risk among adults in Denmark: a population-based case-control study. Cancer Causes Control. (2021) 32:935–42. 10.1007/s10552-021-01446-w 34050843

[13] Veldman J Visser L Huberts-Kregel M Muller N Hepkema B van den Berg A. Rosetting T cells in Hodgkin lymphoma are activated by immunological synapse components HLA class II and CD58. Blood. (2020) 136:2437–41. 10.1182/blood.2020005546 32589698 PMC7685209

[14] Kim HJ Ko YH Kim JE Lee SS Lee H Park G. Epstein-barr virus-associated lymphoproliferative disorders: review and update on 2016 WHO classification. J Pathol Transl Med. (2017) 51:352–8. 10.4132/jptm.2017.03.15 28592786 PMC5525035

[15] Liu W.R. Shipp M.A.. Signaling pathways and immune evasion mechanisms in classical Hodgkin lymphoma. Blood. (2017) 130:2265–70. 10.1182/blood-2017-06-781989 29167175 PMC5701523

[16] Gordon SR Maute RL Dulken BW Hutter G George BM McCracken MN. PD-1 expression by tumour-associated macrophages inhibits phagocytosis and tumour immunity. Nature. (2017) 545:495–9. 10.1038/nature22396 28514441 PMC5931375

[17] Baumeister SH Freeman GJ Dranoff G Sharpe AH. Coinhibitory Pathways in Immunotherapy for Cancer. In: Annual Review of Immunology, Vol 34, Littman DR Yokoyama WM. Editors. (2016) p. 539–573. 10.1146/annurev-immunol-032414-112049 26927206

[18] Henry M Buck S Savaşan S. Flow cytometry for assessment of the tumor microenvironment in pediatric Hodgkin lymphoma. Pediatr Blood Cancer. (2018) 65:e27307. 10.1002/pbc.27307 30009533 PMC6854677

[19] Herrera AF Palmer J Adhikarla V Yamauchi D Poku EK Bading J. Anti-CD25 radioimmunotherapy with BEAM autologous hematopoietic cell transplantation conditioning in Hodgkin lymphoma. Blood Adv. (2021) 5:5300–11. 10.1182/bloodadvances.2021004981 34638132 PMC9153018

[20] Visser L Rutgers B Diepstra A van den Berg A Sattarzadeh A. Characterization of the Microenvironment of Nodular Lymphocyte Predominant Hodgkin Lymphoma. Int J Mol Sci. (2016) 17:2127. 10.3390/ijms17122127 27999289 PMC5187927

[21] Swerdlow SH Campo E Harris NL Jaffe ES Pileri SA Stein H. editors. WHO Classification of Tumors of Haematopoietic Lymphoid Tissues. Edn. Revised 4th. Lyon France: International Agency for Research on Cancer. (2017). Available online at: https://publications.iarc.fr/Book-And-Report-Series/Who-Classification-Of-Tumours/WHO-Classification-Of-Tumours-Of-Haematopoietic-And-Lymphoid-Tissues-2017

[22] Diehl V Thomas RK Re D. Part II: Hodgkin's lymphoma—diagnosis and treatment. The Lancet Oncol. (2004) 5:19–26. 10.1016/S1470-2045(03)01320-2 14700605

[23] Ansell SM. Hodgkin lymphoma: 2018 update on diagnosis, risk-stratification, and management. Am J Hematol. (2018) 93:704–15. 10.1002/ajh.25071 29634090

[24] Engert A Eichenauer DA Dreyling M Grp EGW. Hodgkin's lymphoma: ESMO Clinical Practice Guidelines for diagnosis, treatment and follow-up. Annals Oncol. (2010) 21:v168–v171. 10.1093/annonc/mdq181 20555072

[25] Mauz-Korholz C Lange T Hasenclever D Burkhardt B Feller AC Dorffel W.. Pediatric nodular lymphocyte-predominant hodgkin lymphoma: treatment recommendations of the GPOH-HD study group. Klin Padiatr. (2015) 227:314–21. 10.1055/s-0035-1559664 26356319

[26] Cheson BD Fisher RI Barrington SF Cavalli F Schwartz LH Zucca E. Recommendations for initial evaluation, staging, and response assessment of Hodgkin and non-Hodgkin lymphoma: the Lugano classification. J Clin Oncol. (2014) 32:3059–68. 10.1200/JCO.2013.54.8800 25113753 PMC4979083

[27] Pericart S Tosolini M Gravelle P Rossi C Traverse-Glehen A Amara N. Profiling immune escape in Hodgkin's and diffuse large B-cell lymphomas using the transcriptome and immunostaining. Cancers. (2018) 10:415. 10.3390/cancers10110415 30384489 PMC6266061

[28] Piris MA Medeiros LJ Chang KC. Hodgkin lymphoma: a review of pathological features and recent advances in pathogenesis. Pathology. (2020) 52:154–65. 10.1016/j.pathol.2019.09.005 31699300

[29] Marshall NA Culligan DJ Tighe J Johnston PW Barker RN Vickers MA. The relationships between Epstein-Barr virus latent membrane protein 1 and regulatory T cells in Hodgkin's lymphoma. Exp Hematol. (2007) 35:596–604. 10.1016/j.exphem.2007.01.030 17379070

[30] Küppers R Rajewsky K. The origin of Hodgkin and Reed/Sternberg cells in Hodgkin's disease. Annu Rev Immunol. (1998) 16:471–93. 10.1146/annurev.immunol.16.1.471 9597138

[31] Nagasaki J Togashi Y Sugawara T Itami M Yamauchi N Yuda J. The critical role of CD4+ T cells in PD-1 blockade against MHC-II-expressing tumors such as classic Hodgkin lymphoma. Blood Adv. (2020) 4:4069–82. 10.1182/bloodadvances.2020002098 32870971 PMC7479950

[32] Brunner-Weinzierl MC Rudd CE. CTLA-4 and PD-1 control of T-Cell motility and migration: implications for tumor immunotherapy. Front Immunol. (2018) 9:2737. 10.3389/fimmu.2018.02737 30542345 PMC6277866

[33] Roemer MGM Redd RA Cader FZ Pak CJ Abdelrahman S Ouyang J. Major histocompatibility complex class II and programmed death ligand 1 expression predict outcome after programmed death 1 blockade in classic Hodgkin lymphoma. J Clin Oncol. (2018) 36:942–50. 10.1200/JCO.2017.77.3994 29394125 PMC5877802

[34] Cader FZ Schackmann RCJ Hu X Wienand K Redd R Chapuy B. Mass cytometry of Hodgkin lymphoma reveals a CD4(+) regulatory T-cell-rich and exhausted T-effector microenvironment. Blood. (2018) 132:825–36. 10.1182/blood-2018-04-843714 29880615 PMC6107878

[35] Alonso-Alvarez S Vidriales MB Caballero MD Blanco O Puig N Martin A. The number of tumor infiltrating T-cell subsets in lymph nodes from patients with Hodgkin lymphoma is associated with the outcome after first line ABVD therapy. Leuk Lymphoma. (2017) 58:1144–52. 10.1080/10428194.2016.1239263 27733075

[36] Machicote A Belen S Baz P Billordo LA Fainboim L. Human CD8(+)HLA-DR(+) Regulatory T cells, similarly to classical CD4(+)Foxp3(+) cells, suppress immune responses via PD-1/PD-L1 axis. Front Immunol. (2018) 9:2788. 10.3389/fimmu.2018.02788 30555473 PMC6281883

[37] Buchbinder E.I. Desai A.. CTLA-4 and PD-1 pathways: similarities, differences, and implications of their inhibition. Am J Clin Oncol. (2016) 39:98–106. 10.1097/COC.0000000000000239 26558876 PMC4892769

[38] Vardhana S Younes A.. The immune microenvironment in Hodgkin lymphoma: T cells, B cells, immune checkpoints. Haematologica. (2016) 101:794–802. 10.3324/haematol.2015.132761 27365459 PMC5004458

[39] Steidl C Shah SP Woolcock BW Rui L Kawahara M Farinha P. MHC class II transactivator CIITA is a recurrent gene fusion partner in lymphoid cancers. Nature. (2011) 471:377–81. 10.1038/nature09754 21368758 PMC3902849

[40] Hollander P Kamper P Smedby KE Enblad G Ludvigsen M Mortensen J. High proportions of PD-1(+) and PD-L1(+) leukocytes in classical Hodgkin lymphoma microenvironment are associated with inferior outcome. Blood Adv. (2017) 1:1427–39. 10.1182/bloodadvances.2017006346 29296784 PMC5727849

[41] Váróczy L Gergely L Miltényi Z Aleksza M Illés A. Can CD3+/HLA-DR+ activated T cells predict the prognosis of non-Hodgkin's lymphoma patients? Immunol Lett. (2005) 97:155–7. 10.1016/j.imlet.2004.10.005 15626488

[42] Ohno Y Kitamura H Takahashi N Ohtake J Kaneumi S Sumida K. IL-6 down-regulates HLA class II expression and IL-12 production of human dendritic cells to impair activation of antigen-specific CD4(+) T cells. Cancer Immunol Immunother. (2016) 65:193–204. 10.1007/s00262-015-1791-4 26759006 PMC11028987

[43] Kitamura H Ohno Y Toyoshima Y Ohtake J Homma S Kawamura H. Interleukin-6/STAT3 signaling as a promising target to improve the efficacy of cancer immunotherapy. Cancer Sci. (2017) 108:1947–52. 10.1111/cas.13332 28749573 PMC5623748

[44] Morinobu A Gadina M Strober W Visconti R Fornace A Montagna C. STAT4 serine phosphorylation is critical for IL-12-induced IFN-gamma production but not for cell proliferation. Proc Natl Acad Sci U S A. (2002) 99:12281–6. 10.1073/pnas.182618999 12213961 PMC129436

[45] Bertuzzi C Sabattini E Agostinelli C. Immune microenvironment features and dynamics in Hodgkin lymphoma. Cancers (Basel). (2021) 13:3634. 10.3390/cancers13143634 34298847 PMC8304929

[46] Taylor J.G. Gribben J.G.. Microenvironment abnormalities and lymphomagenesis: Immunological aspects. Semin Cancer Biol. (2015) 34:36–45. 10.1016/j.semcancer.2015.07.004 26232774 PMC4592463

[47] Cohen M Vistarop AG Huaman F Narbaitz M Metrebian F De Matteo E. Cytotoxic response against Epstein Barr virus coexists with diffuse large B-cell lymphoma tolerogenic microenvironment: clinical features and survival impact. Sci Rep. (2017) 7:10813. 10.1038/s41598-017-11052-z 28883511 PMC5589929

