# Supplementary material for: High percentages of peripheral blood T-cell activation in childhood Hodgkin's lymphoma are associated with inferior outcome
Source: Front Med (Lausanne). 2022 Aug 10;9:955373. doi: 10.3389/fmed.2022.955373 (PMC9399494; doi:10.3389/fmed.2022.955373)
Supplement: Supplementary file 1 [file Image_1.pdf]

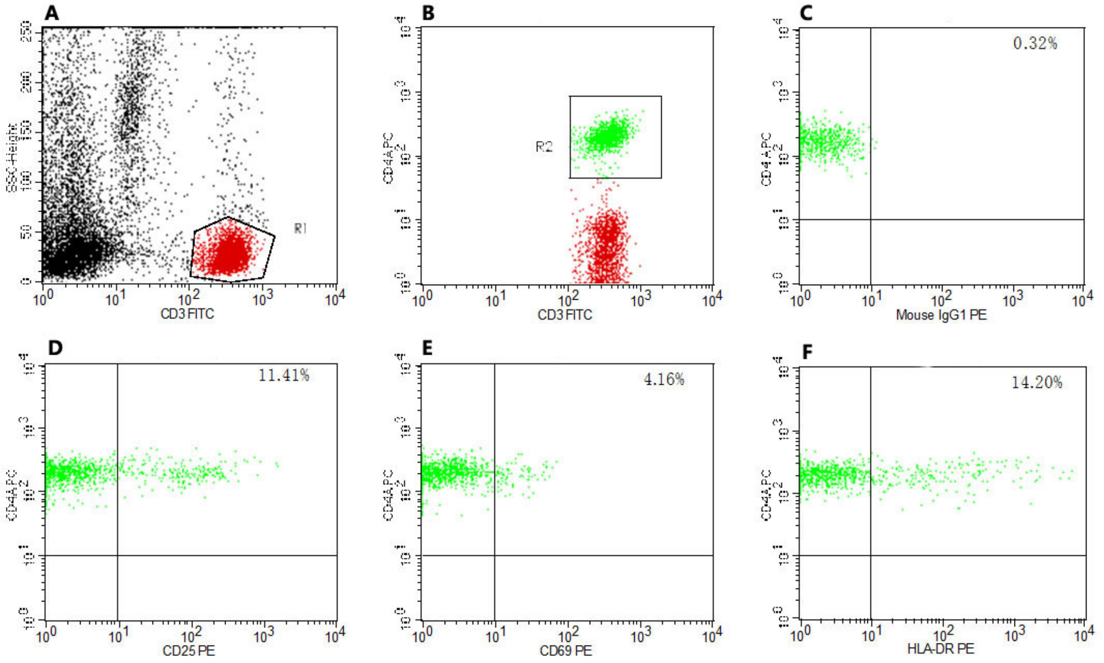

Supplementary Figure 1. Flow cytometric analysis diagrams of peripheral blood with HL. **A,** Biparametric dot plot showing side scatter (SSC) and CD3 expression (R1); **B,** Flow cytometric diagram for demonstrating a certain population of cells with CD3+CD4+ co-expression in the upper right quadrant (green spots, R2). **C,** flow cytometry showing a mouse IgG Isotype control; **D-F**, Flow cytometry detected a population of cells in upper right quadrant with CD3+CD4+CD25+ (11.41%), CD3+CD4+CD69+ (4.16%) and CD3+CD4+HLA-DR+ (14.20%) T lymphocytes refer to R2, respectively.

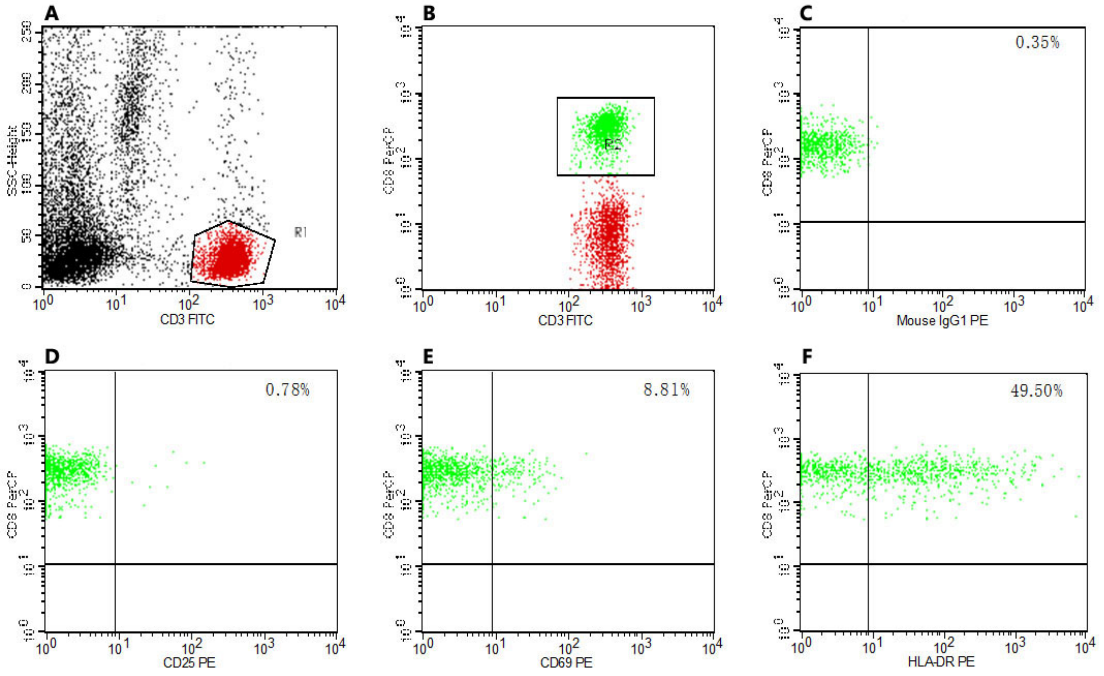

Supplementary Figure 2. Flow cytometric analysis diagrams of peripheral

blood with HL. **A,** Biparametric dot plot showing side scatter (SSC) and CD3 expression (R1); **B,** Flow cytometric diagram for demonstrating a certain population of cells with CD3+CD8+ co-expression in the upper right quadrant (green spots, R2). **C,** flow cytometry showing a mouse IgG Isotype control; **D-F**, Flow cytometry detected a population of cells in upper right quadrant with CD3+CD8+CD25+ (0.78%), CD3+CD8+CD69+ (8.81%) and CD3+CD8+HLA-DR+ (49.50%) T lymphocytes refer to R2, respectively.
